# Supplementary material for: MerlinS13 phosphorylation regulates meningioma Wnt signaling and magnetic resonance imaging features
Source: Nat Commun. 2024 Sep 9;15:7873. doi: 10.1038/s41467-024-52284-8 (PMC11383945; doi:10.1038/s41467-024-52284-8)
Supplement: Supplementary file 1 — Supplementary Information [file 41467_2024_52284_MOESM1_ESM.pdf]

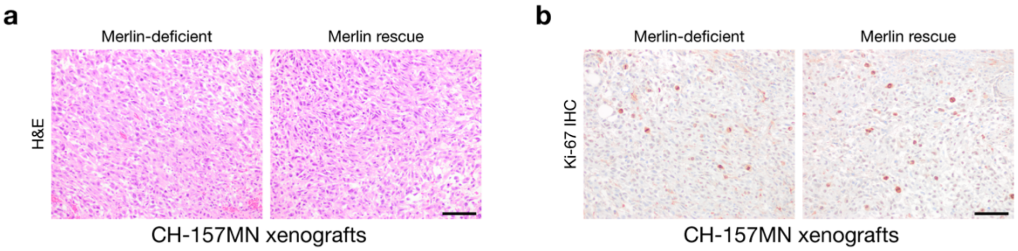

Supplementary Fig. 1. CH-157MN xenograft model of Merlin-deficient or Merlin rescue meningiomas. a-b, Images of H&E staining **a** or Ki-67 immunohistochemistry **b** from CH-157MN meningioma xenografts in NU/NU mice with or without doxycycline-inducible Merlin-rescue. Images are representative of n=3 xenografts/condition. Scale bars, 100 μm. Source data are provided as a Source Data file.

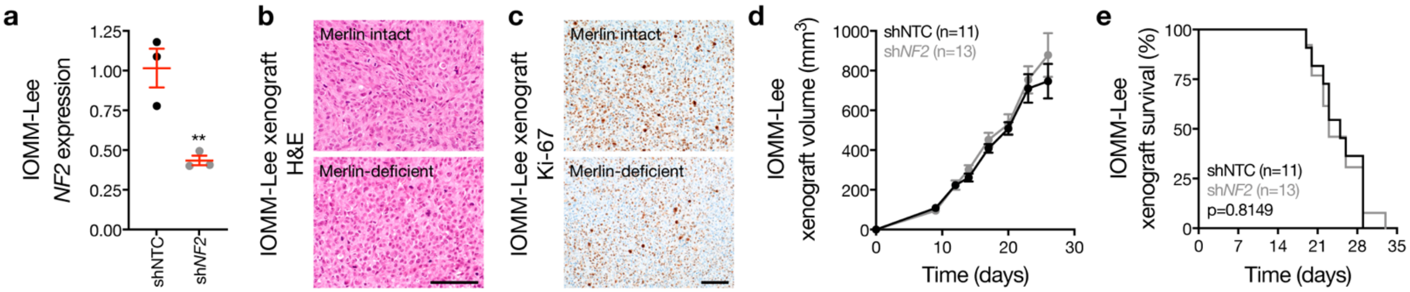

Supplementary Fig. 2. IOMM-Lee xenograft model of Merlin intact or Merlin-deficient meningiomas. a, QPCR for *NF2* in IOMM-Lee meningioma cells expressing non-targeted control shRNAs (shNTC) or shRNAs suppressing *NF2*. n=3 biological replicates per condition. **b-c**, Images of H&E staining **b** or Ki-67 immunohistochemistry **c** from IOMM-Lee meningioma xenografts in NU/NU mice with or without shRNA suppression of *NF2*. Images are representative of n=3 xenografts/condition. Scale bars, 100 µm. **d**, IOMM-Lee xenograft measurements in NU/NU mice with or without shRNA suppression of *NF2*. n=12 mice with shNTC and 13 mice with sh*NF2*. **e**, Kaplan-Meier survival curve for IOMM-Lee xenograft overall survival in NU/NU mice as in **d** (log-rank test). n=12 mice with shNTC and 13 mice with sh*NF2*. Lines represent means, and error bar represent standard error of the means. **P≤0.01 (Student's t-test, one sided). Source data are provided as a Source Data file.

a

Sample number	Sample identifier	Condition	C1	C2	C3	C4	C5	C6	C7	C8	C9	C10	C11	C12	C13	C14	C15	C16	C17	Total
1	241_10	Merlin-deficient	669	323	482	344	480	478	578	302	654	162	318	233	33	55	63	76	78	5328
3	241_3	Merlin-deficient	380	461	378	335	416	420	377	307	270	250	203	186	50	62	64	35	26	4220
4	241_4	Merlin-deficient	97	352	124	150	78	157	168	183	181	74	96	30	82	93	32	12	16	1925
8	287_2	Merlin-deficient	149	230	124	195	56	120	52	172	83	55	103	52	36	31	7	23	7	1495
9	287_3	Merlin-deficient	290	213	228	262	120	147	130	158	98	44	81	48	57	34	14	24	10	1958
2	241_2	Merlin rescue	259	137	272	152	170	110	153	88	100	200	87	81	3	12	11	7	14	1856
5	241_5	Merlin rescue	588	385	550	417	602	570	634	405	380	291	219	370	54	17	65	47	61	5655
6	241_6	Merlin rescue	663	840	440	809	488	605	603	545	330	249	202	293	172	39	55	45	43	6421
7	241_8	Merlin rescue	482	711	519	327	377	364	337	459	369	171	259	147	57	137	50	57	35	4858
10	287_4	Merlin rescue	402	265	252	246	382	182	171	146	145	70	134	95	119	69	35	29	19	2761
11	287_5	Merlin rescue	147	102	160	119	116	87	53	64	137	27	70	38	10	64	12	18	9	1233
12	287_6	Merlin rescue	384	243	180	299	233	184	147	161	129	664	86	120	103	48	19	42	13	3055
		**Total**	4510	4262	3709	3655	3518	3424	3403	2990	2876	2257	1858	1693	776	661	427	415	331	40765

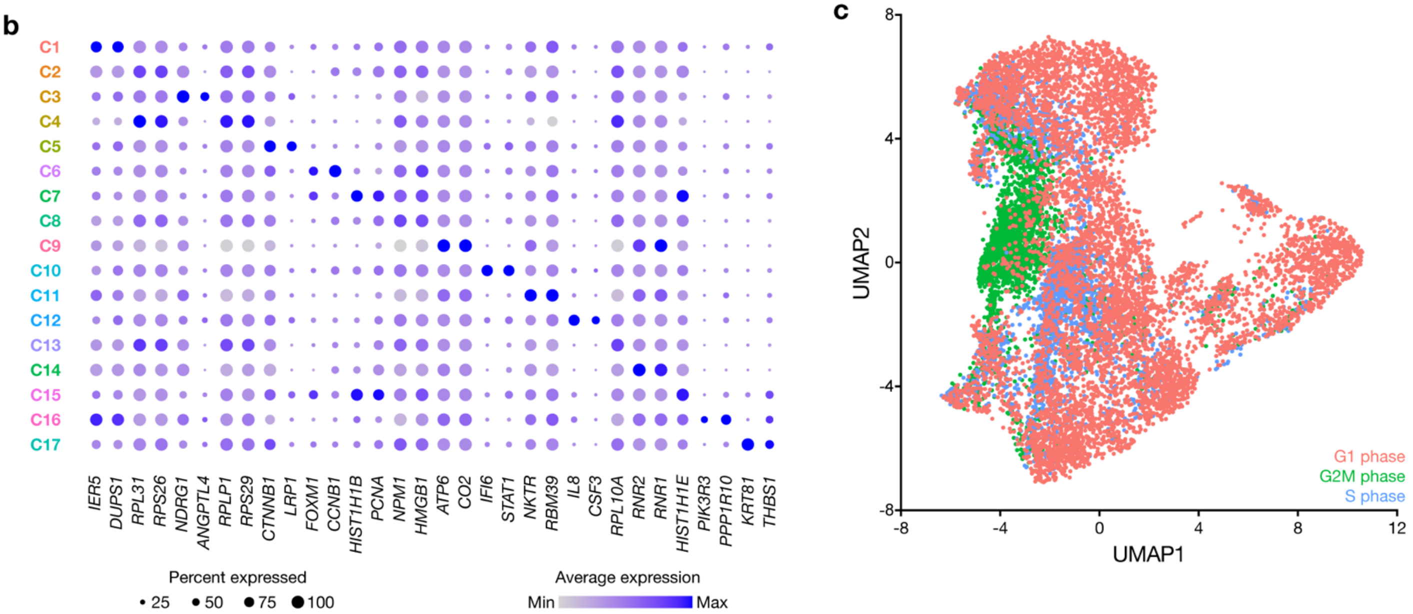

Supplementary Fig. 3. Single-cell RNA sequencing of CH-157MN xenograft models of Merlin-deficient or Merlin rescue meningiomas. a, Single-cell RNA sequencing of CH-157MN meningioma xenografts in NU/NU mice with or without Merlin rescue identifies 17 cell states. Table shows number of cells per sample contributing to each cell state colored by assignments from Louvain clustering. **b**, Dot plot of single-cell RNA sequencing showing marker genes for each cell state cluster. **c**, Uniform manifold approximation and projection (UMAP) of single-cell RNA sequencing transcriptomes of 40,765 CH-157MN cells from 12 xenografts as in **Fig. 1a-c** showing cell cycle distribution. Source data are provided as a Source Data file.

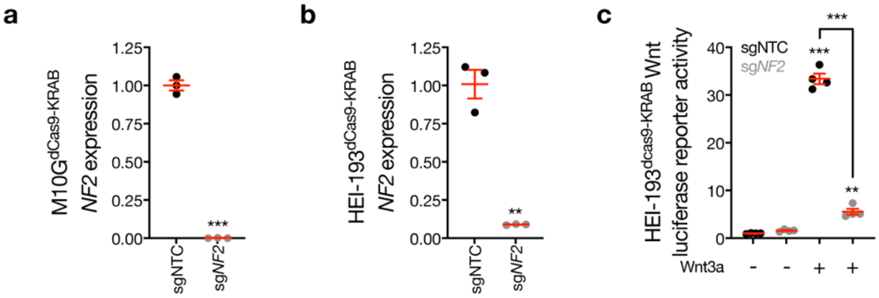

Supplementary Fig. 4. Merlin suppression inhibits Wnt signaling. a, QPCR for *NF2* in M10G$^{dCas9\text{-}KRAB}$ meningioma cells expressing non-targeted control sgRNAs (sgNTC) or sgRNAs suppressing *NF2* (sg*NF2*). n=3 biological replicates per condition. **b**, QPCR for *NF2* in HEI-193$^{dCas9\text{-}KRAB}$ schwannoma cells expressing sgNTC or sg*NF2*. n=3 biological replicates per condition. **c**, TOP-Flash Tcf/Lef luciferase reporter assay in HEI-193$^{dCas9\text{-}KRAB}$ schwannoma cells expressing sgNTC or sg*NF2* with or without 24-hours of Wnt3a treatment (100ng/µl). n=4 biological replicates per condition. Lines represent means, and error bar represent standard error of the means. **P≤0.01, ***P≤0.0001 (Student's t-test, one sided). Source data are provided as a Source Data file.

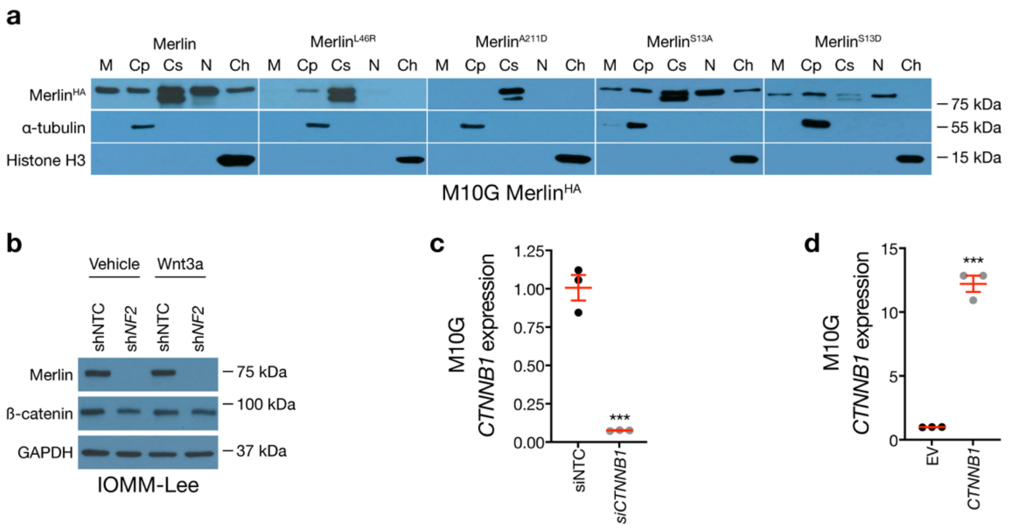

Supplementary Fig. 5. Genetic perturbation of Merlin or β-catenin in meningioma cells. a, Immunoblots for HA (Merlin) after biochemical fractionation of M10G meningioma cells overexpressing Merlin constructs. Immunoblots for α-tubulin or histone H3 mark cytoplasmic or chromatin fractions, respectively. Representative of 4 biological replicates. **b**, Immunoblots for Merlin, β-catenin, or GAPDH in IOMM-Lee meningioma cells expressing non-targeted control shRNAs (shNTC) or shRNAs suppressing *NF2* (sh*NF2*) with or without 24-hours of Wnt3a treatment (100ng/ul). Representative of 3 biological replicates. **c**, QPCR for β-catenin (*CTNNB1*) in M10G meningioma cells expressing non-targeted control siRNAs (siNTC) or siRNAs suppressing β-catenin (si*CTNNB1*). n=3 biological replicates per condition. **d**, QPCR for β-catenin (*CTNNB1*) in M10G meningioma cells with or without β-catenin overexpression. n=3 biological replicates per condition. Lines represent means, and error bar represent standard error of the means. ***P≤0.0001 (Student's t-test, one sided). Source data are provided as a Source Data file.

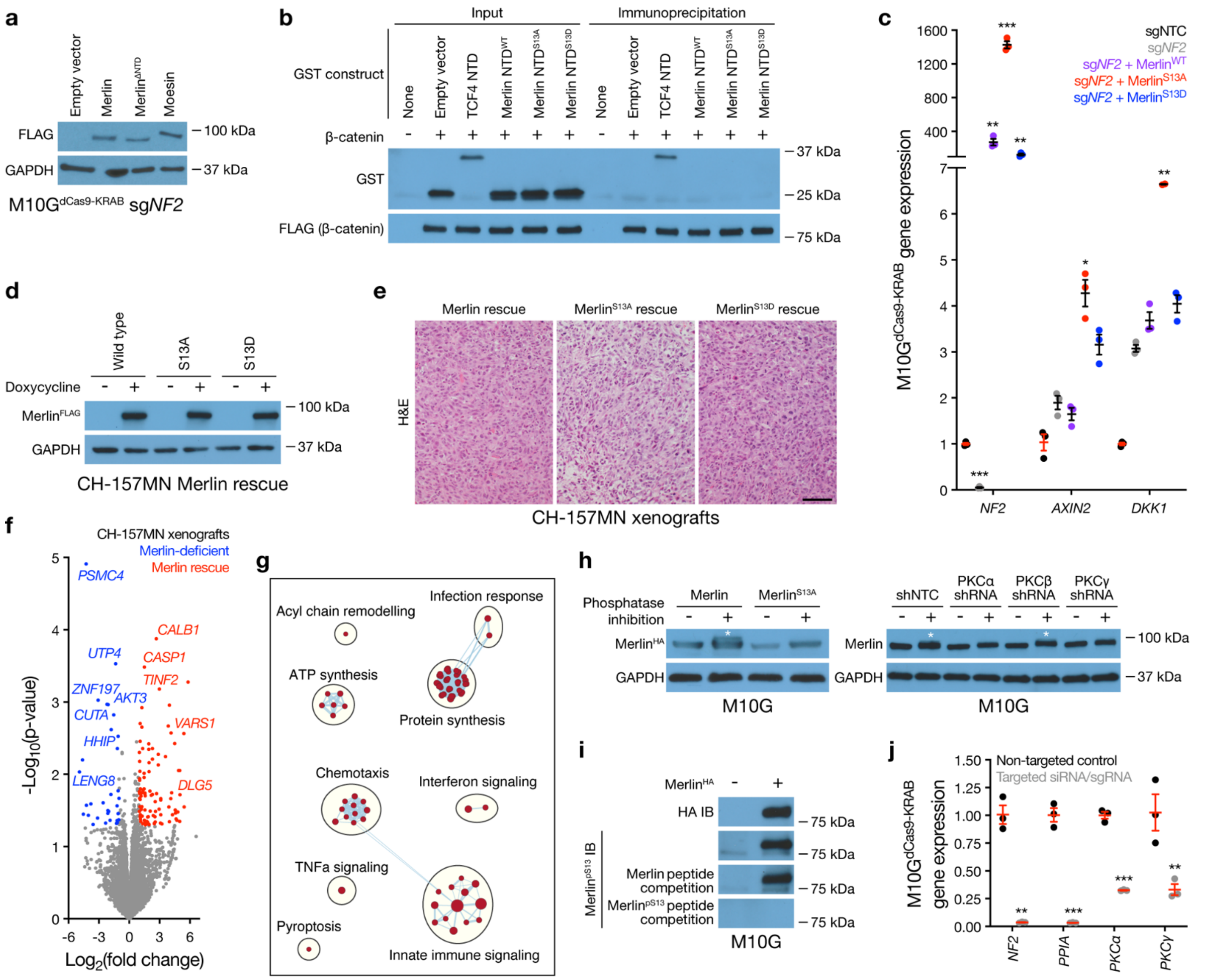

Supplementary Fig. 6. PKC and PP1A regulate Merlin Serine 13 phosphorylation. **a**, Immunoblots for FLAG (Merlin constructs or Moesin) or GAPDH in M10G$^{dCas9\text{-}KRAB}$ meningioma cells expressing sgRNAs suppressing *NF2* (sg*NF2*) with or without rescue of Merlin constructs or overexpression of the FERM family member Moesin. Representative of 3 biological replicates. **b**, *In vitro* recombinant protein binding assay between truncated GST constructs (top immunoblot) and full-length β-catenin with a FLAG tag (bottom immunoblot). The 54 residues of the TCF4 N-terminal domain (NTD) were used as a positive control for binding to β-catenin, and conditions without prey protein or GST only (Empty vector) were used as negative controls. Result show that the 19 residues of the Merlin NTD are not sufficient for binding to β-catenin, irrespective of unphosphorylatable or phosphomimetic substitutions at S13. These data suggest that the tertiary structure of Merlin (or intermediate proteins that may facilitate interaction) may be necessary for binding to β-catenin. Representative of 3 biological replicates. **c**, QPCR for the genes indicated on the x-axis in M10G$^{dCas9\text{-}KRAB}$ meningioma cells expressing non-targeted control sgRNAs (sgNTC), sgRNAs suppressing *NF2* (sg*NF2*), or sg*NF2* with rescue of Merlin wildtype (WT), S13A, or S13D constructs. Significance is shown compared to sgNTC for *NF2* expression across all conditions or compared to all other conditions for *AXIN1* or *DKK1* expression with S13A rescue. n=4 biological replicates per condition. **d**, Immunoblots for FLAG (Merlin) or GAPDH in CH-157MN meningioma cells with or without 24-hours of doxycycline-inducible Merlin rescue (20µg/ml). Representative of 3 biological replicates. **e**, Images of H&E staining CH-157MN xenografts in NU/NU mice with doxycycline-inducible Merlin-rescue. Images are representative of n=3 xenografts/condition. Scale bars, 100 µm. **f**, Volcano plot showing differentially expressed genes from bulk RNA sequencing of CH-157MN meningioma xenografts without (blue, n=3) versus with (red, n=4) doxycycline-inducible Merlin rescue. **g**, Network of gene circuits distinguishing CH-157MN meningioma xenografts with (n=4) versus without (n=3) doxycycline-inducible Merlin rescue using RNA sequencing. Nodes represent pathways and edges represent shared genes between pathways (P≤0.01,

FDR≤0.01). **h**, Immunoblots of HA (Merlin), endogenous Merlin or GAPDH in M10G meningioma cells with or without (1) phosphatase inhibition, (2) doxycycline-inducible Merlin overexpression, or (3) expression of non-targeted control shRNAs (shNTC) or shRNAs suppressing PKC isoforms. Asterisks show doublets suggestive of phosphorylation events. Representative of 3 biological replicates. **i**, Immunoblots (IB) for HA (Merlin, top) or Merlin S13 phosphorylation (Merlin^{pS13}) after Merlin overexpression. The bottom three immunoblots were incubated in 4µg primary phospho-specific Merlin S13 antibody with or without spike-in of synthetic Merlin peptides used for rabbit immunization during phospho-specific antibody generation. Antibody incubations with no peptide (second from top), phospho-peptides (bottom blot, CSRMSFS(pS)LKRKQP-amide, 20µg) or unphosphorylated peptides (second to bottom blot, CSRMSFSSLKRKQP-amide, 20µg) show competition for Merlin^{pS13} binding only when Merlin^{pS13} antibody is incubated with phosphorylated peptide. Representative of 3 biological replicates. **j**, QPCR for the genes indicated on the x-axis in M10G$^{dCas9\text{-}KRAB}$ meningioma cells expressing non-targeted control sgRNAs or siRNAs, sgRNAs suppressing *NF2* (sg*NF2*, first pair), or siRNAs suppressing PP1A or PKC isoforms (second through fourth pairs). Expression of genes of interest in experimental conditions were compared to appropriate non-targeted control sgRNAs (for *NF2* suppression using sgRNAs) or siRNAs (for PP1A and PKC isoform suppression using siRNAs). n=3 biological replicates per condition. Lines represent means, and error bar represent standard error of the means. *P≤0.05, **P≤0.01, ***P≤0.0001 (Student's t-test, one sided). Source data are provided as a Source Data file.

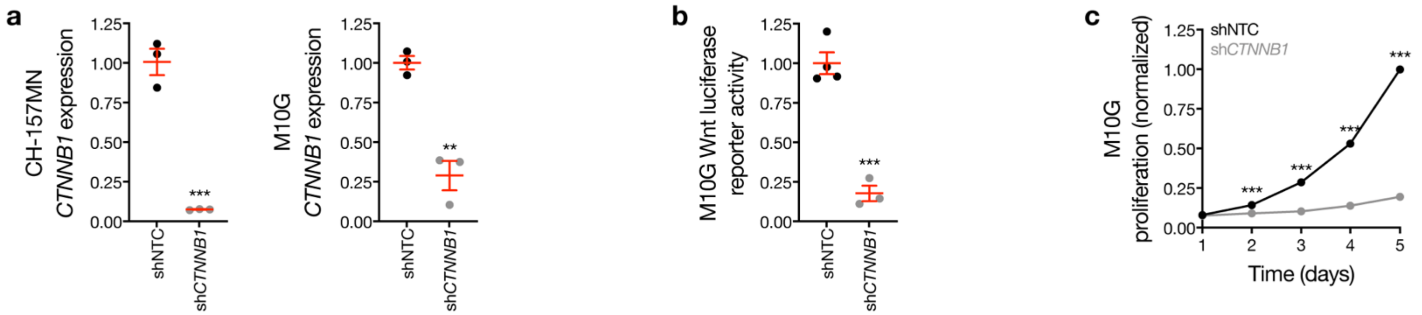

Supplementary Fig. 7. Wnt pathway inhibition blocks meningioma cell proliferation. a, QPCR for β-catenin (*CTNNB1*) in CH-157MN or M10G meningioma cells expressing non-targeted control shRNAs (shNTC) or shRNAs suppressing β-catenin (sh*CTNNB1*). **b**, TOP-Flash Tcf/Lef luciferase reporter assay in M10G cells expressing shNTC or sh*CTNNB1* with or without 24-hours of Wnt3a treatment (100ng/µl). **c**, M10G cell MTT assays for cell proliferation with shNTC or sh*CTNNB1* expression. Lines represent means, and error bar represent standard error of the means. **P≤0.01, ***P≤0.0001 (Student's t-test, one sided). n=3 biological replicates per condition in all panels. Source data are provided as a Source Data file.
