# Supplementary material for: MerlinS13 phosphorylation regulates meningioma Wnt signaling and magnetic resonance imaging features
Source: Nat Commun. 2024 Sep 9;15:7873. doi: 10.1038/s41467-024-52284-8 (PMC11383945; doi:10.1038/s41467-024-52284-8)
Supplement: Supplementary file 3 — Description of Additional Supplementary Files [file 41467_2024_52284_MOESM3_ESM.pdf]

Description of Additional Supplementary Files

Supplementary Data 1. CH-157MN meningioma xenograft single-cell RNA sequencing.

Supplementary Data 2. Merlin-APEX2 proximity-labeling proteomic mass spectrometry in M10G meningioma cells.

Supplementary Data 3. CH-157MN meningioma xenograft bulk RNA sequencing.

Supplementary Data 4. Wnt target gene expression across Merlin-intact human meningiomas.
