# Supplementary material for: MerlinS13 phosphorylation regulates meningioma Wnt signaling and magnetic resonance imaging features
Source: Nat Commun. 2024 Sep 9;15:7873. doi: 10.1038/s41467-024-52284-8 (PMC11383945; doi:10.1038/s41467-024-52284-8)
Supplement: Supplementary file 5 — Reporting Summary [file 41467_2024_52284_MOESM5_ESM.pdf]

nature portfolio

Corresponding author(s): David Raleigh
Last updated by author(s): Aug 20, 2024

Reporting Summary

Nature Portfolio wishes to improve the reproducibility of the work that we publish. This form provides structure for consistency and transparency in reporting. For further information on Nature Portfolio policies, see our Editorial Policies and the Editorial Policy Checklist.

Statistics

For all statistical analyses, confirm that the following items are present in the figure legend, table legend, main text, or Methods section.

n/a	Confirmed	
☐	☒	The exact sample size (n) for each experimental group/condition, given as a discrete number and unit of measurement
☐	☒	A statement on whether measurements were taken from distinct samples or whether the same sample was measured repeatedly
☐	☒	The statistical test(s) used AND whether they are one- or two-sided<br>*Only common tests should be described solely by name; describe more complex techniques in the Methods section.*
☐	☒	A description of all covariates tested
☐	☒	A description of any assumptions or corrections, such as tests of normality and adjustment for multiple comparisons
☐	☒	A full description of the statistical parameters including central tendency (e.g. means) or other basic estimates (e.g. regression coefficient) AND variation (e.g. standard deviation) or associated estimates of uncertainty (e.g. confidence intervals)
☐	☒	For null hypothesis testing, the test statistic (e.g. F, t, r) with confidence intervals, effect sizes, degrees of freedom and P value noted<br>*Give P values as exact values whenever suitable.*
☒	☐	For Bayesian analysis, information on the choice of priors and Markov chain Monte Carlo settings
☐	☒	For hierarchical and complex designs, identification of the appropriate level for tests and full reporting of outcomes
☐	☒	Estimates of effect sizes (e.g. Cohen's d, Pearson's r), indicating how they were calculated

Our web collection on statistics for biologists contains articles on many of the points above.

Software and code

Policy information about availability of computer code

Data collection	No software for data collection
Data analysis	Cell Ranger version v6.1.2 (10X Genomics), Seurat version v4.3.0, R Studio v4.2.1. The code used for this study can be found at: https://github.com/cdeaton380/Merlin-rescue. Quality control and preprocessing: FastQC v0.11.9, MultiQC v1.12, Cutadapt v3.7. Read alignment and quantification: HISAT2 v2.2.0, featureCounts v2.0.3 from the Subread package. Differential expression analysis: R v4.3.1, DESeq2 v1.36.0, with ggplot2 and pheatmap for visualization. Pathway analysis: GSEA v4.3.2, Cytoscape v3.10.0 with EnrichmentMap App v3.3.6 and AutoAnnotate app v1.4.1.

For manuscripts utilizing custom algorithms or software that are central to the research but not yet described in published literature, software must be made available to editors and reviewers. We strongly encourage code deposition in a community repository (e.g. GitHub). See the Nature Portfolio guidelines for submitting code & software for further information.

Data

Policy information about availability of data

All manuscripts must include a data availability statement. This statement should provide the following information, where applicable:
- Accession codes, unique identifiers, or web links for publicly available datasets
- A description of any restrictions on data availability
- For clinical datasets or third party data, please ensure that the statement adheres to our policy

All raw data for all newly generated datasets reported in this manuscript have been deposited. Single-cell RNA sequencing reported in this manuscript have been

deposited in the NCBI Gene Expression Omnibus under the accession GSE224347 [https://www.ncbi.nlm.nih.gov/geo/query/acc.cgi?acc=GSE224347]. Transcriptomes were simultaneously aligned against publicly available hg19 and mm10 data sets, stored on the UCSF C4 environment at refdata-cellranger-hg19-and-mm10-3.0.0/refdata-cellranger-mm10-3.0.0/. Cells with transcripts aligned to <99% of the human dataset were discarded. Bulk RNA sequencing data reported in this manuscript has been deposited in the BioProject database under BioProject ID PRJNA1102120 [https://www.ncbi.nlm.nih.gov/bioproject/?term=PRJNA1102120]. Proximity labeling proteomic mass spectrometry data reported in this manuscript have been deposited to PRIDE Proteomics Identification database under the accession 36993679 (https://www.ebi.ac.uk/pride/archive/projects/PXD053578). The remaining data are available within the Article, Supplementary Information or Source Data file. Source data are provided with this paper.

Research involving human participants, their data, or biological material

Policy information about studies with human participants or human data. See also policy information about sex, gender (identity/presentation), and sexual orientation and race, ethnicity and racism.

Reporting on sex and gender	The clinical samples used in this study were retrospective and nonrandomized with no intervention, and all samples were interrogated equally. Thus, controlling for covariates among clinical samples is not relevant.
Reporting on race, ethnicity, or other socially relevant groupings	NA
Population characteristics	The clinical samples used in this study were retrospective and nonrandomized with no intervention, and all samples were interrogated equally. Thus, controlling for covariates among clinical samples is not relevant.
Recruitment	Patients undergoing resection of meningioma who gave consent for research were included in this study. All patients who undergo craniotomy for tumor resection at UCSF sign consent to provide tissue for research. Thus, there was no self-selection bias or other biases that may influence or impact our results.
Ethics oversight	This study complied with all relevant ethical regulations and was approved by the UCSF Institutional Review Board (13-12587, 17-22324, 17-23196 and 18-24633).

Note that full information on the approval of the study protocol must also be provided in the manuscript.

Field-specific reporting

Please select the one below that is the best fit for your research. If you are not sure, read the appropriate sections before making your selection.

☒ Life sciences ☐ Behavioural & social sciences ☐ Ecological, evolutionary & environmental sciences

For a reference copy of the document with all sections, see nature.com/documents/nr-reporting-summary-flat.pdf

Life sciences study design

All studies must disclose on these points even when the disclosure is negative.

Sample size	Functional experiments: individual replicates were performed in at least triplicates.<br>scRNA seq: we provide 5-7 replicates for each condition used, between two biological repeats to control for batch differences<br>BulkRNA seq: we provide 3-4 replicates for each condition.<br>phospho-proteomic MS: performed with technical triplicates<br>APEX proximity labeling: performed with technical triplicates
Data exclusions	No data was excluded
Replication	scRNA seq was repeated over two biological replicates<br>functional data was repeated and reproducible<br>We used 3-4 biological replicates for bulk-RNAseq
Randomization	Not a randomised study
Blinding	MRI imaging was performed by collaborating group without prior knowledge of groupings. Collaborators were blinded by labelling samples as numbers instead of sample names.

Reporting for specific materials, systems and methods

We require information from authors about some types of materials, experimental systems and methods used in many studies. Here, indicate whether each material, system or method listed is relevant to your study. If you are not sure if a list item applies to your research, read the appropriate section before selecting a response.

Materials & experimental systems

n/a	Involved in the study	
☐	☒	Antibodies
☐	☒	Eukaryotic cell lines
☒	☐	Palaeontology and archaeology
☐	☒	Animals and other organisms
☐	☒	Clinical data
☒	☐	Dual use research of concern
☒	☐	Plants

Methods

n/a	Involved in the study	
☒	☐	ChIP-seq
☒	☐	Flow cytometry
☐	☒	MRI-based neuroimaging

Antibodies

Antibodies used

FLAG: Sigma-Aldrich, Cat# F7425 (1:500-1:2000)
DYKDDDDK: Cell Signaling, Cat# 14793S (1:2000)
HA: Cell Signaling, Cat# 2999S (1:2000)
GAPDH: Thermo Fisher Scientific, Cat# MA515738 (1:5000)
Merlin: Abcam, Cat# ab88957 (1:1000)
α-Tubulin: Sigma-Aldrich, Cat# T5168 (1:5000)
calreticulin: Abcam, Cat# ab92516 (1:10,000)
vimentin: Abcam, Cat# ab8069 (1:10,000)
Rb: Cell Signaling, Cat# 9309S (1:5000)
Histone H3: Thermo, Cat# 702023, (1:5000)-catenin:
BD Biosciences, Cat# 610153 (1:1000)
Anti-phospho-Serine13: a custom antibody developed by Thermo Fisher Scientific using rabbits that were immunized with a synthetic Merlin phospho-peptide sequence (CSRMSFS(pS)LKRKQP-amide) (1:250-:1500)
GST antibody: Cell signaling, Cat# 2622S (1:1000)
anti-mouse conjugated to Alexa Fluor 488 secondary antibody: Thermo Fisher Scientific, Cat# A21202 (1:2000)
streptavidin conjugated to Alexa Fluor 647: Thermo Fisher Scientific, Cat# S21374 (1:2000)
Hoechst: Invitrogen, Cat# H3570 (1:5000)
Ki-67: Ventana, clone 30-9, Cat# 790-4286 (1:6)
Anti-rabbit-HRP: Cell signaling, Cat# 7074 (1:2000)
Anti-mouse-HRP: Cell signaling, Cat# 7076 (1:2000)

Validation

FLAG: validated with and without FLAG over expression
DYKDDDDK: validated with and without overexpression
HA: validated with and without over expression
Merlin: validated in-house with knockdown studies
α-Tubulin: validated in eukaryotes
Calreticulin: knockout validated by manufacturer
vimentin: knockdout validated from manufacturer
Rb: knockout validated by manufacturer
GST: did not bind to the no-GST control-catenin:
kncodkown validated in current study
Anti-phospho-Serine13: validated with peptide competition in current study
anti-mouse conjugated to Alexa Fluor 488: Validated in house with secondary only controls
streptavidin conjugated to Alexa Fluor 647: validated in house with untreated control

Eukaryotic cell lines

Policy information about cell lines and Sex and Gender in Research

Cell line source(s)

CH157MN: human derrived meningioma from female patient
IOMM-Lee: human derrived meningioma from male patient
Hei-193: human derrived schwannoma cell line from unknown sex
M10G: human derrived meningioma cells from female patient

Authentication

Cell lines were STR profiled once per year at the UC Berkeley DNA Sequencing Facility which includes PCR amplification of 9 STR loci plus Amelogenin using Promega GenePrint 10 System, Fragment Analysis with ABI 3730XL DNA Analyzer, comprehensive data analysis with ABI Genemapper software and final verification using supplier databases including ATCC and DSMZ. M10G cells were derrived in house.

Mycoplasma contamination

All cell lines were tested for mycoplasma contamination using the ATCC Universal Mycoplasma Detection kit every 3-4 months. Cell lines used in this project were confirmed mycoplasma free for the duration of this project.

Commonly misidentified lines (See ICLAC register)	none

Animals and other research organisms

Policy information about studies involving animals; ARRIVE guidelines recommended for reporting animal research, and Sex and Gender in Research

Laboratory animals	5-6 week old female NU/NU mice purchased from Harlan Sprague Dawley for this study. All animal care and experimental procedures were in accordance with federal policies and guidelines governing the use of animals and were approved by the University of California San Francisco's (UCSF) Institutional Animal Care and Use Committee (IACUC). The IACUC is in full compliance with the 8th edition of The Guide for the Care and use of Laboratory Animals. UCSF has an AAALAC accredited animal care and use program. Mice were housed in solid-bottomed cages containing autoclaved paper chips in individually ventilated cages. Animals had continuous access to irradiated food and water purified by reverse osmosis and UV lighting. The housing room was maintained at 68 to 74º Fahrenheit with 30-70 % relative humidity. All cages were maintained in a SPF barrier facility from which dirty bedding sentinel mice were tested quarterly. All sentinels were found to be seronegative for mouse hepatitis virus, pneumonia virus of mice, mouse parvovirus, minute virus of mice, epizootic diarrhea of infant mice, Theiler's murine encephalomyelitis virus, ectromelia and were free of ectoparasites and endoparasites. Mice were observed daily by animal care staff for any clinical abnormalities, and were euthanized in accordance with American Veterinary Medical Association (AVMA) guidelines.
Wild animals	none
Reporting on sex	Female NU/NU mice were used. Meningiomas arise in patients of both sexes and the phenotypes reported here do not have differences between sexes in humans, so a decision was made to use female mice to minimize altercations between animals subjects.
Field-collected samples	Study did not involve samples collected in the field.
Ethics oversight	Study was approved by the UCSF Institutional Animal Care and Use Committee (AN174769).

Note that full information on the approval of the study protocol must also be provided in the manuscript.

Clinical data

Policy information about clinical studies

All manuscripts should comply with the ICMJEguidelines for publication of clinical research and a completedCONSORT checklist must be included with all submissions.

Clinical trial registration	*Provide the trial registration number from ClinicalTrials.gov or an equivalent agency.*
Study protocol	*Note where the full trial protocol can be accessed OR if not available, explain why.*
Data collection	*Describe the settings and locales of data collection, noting the time periods of recruitment and data collection.*
Outcomes	*Describe how you pre-defined primary and secondary outcome measures and how you assessed these measures.*

Plants

Seed stocks	*Report on the source of all seed stocks or other plant material used. If applicable, state the seed stock centre and catalogue number. If plant specimens were collected from the field, describe the collection location, date and sampling procedures.*
Novel plant genotypes	*Describe the methods by which all novel plant genotypes were produced. This includes those generated by transgenic approaches, gene editing, chemical/radiation-based mutagenesis and hybridization. For transgenic lines, describe the transformation method, the number of independent lines analyzed and the generation upon which experiments were performed. For gene-edited lines, describe the editor used, the endogenous sequence targeted for editing, the targeting guide RNA sequence (if applicable) and how the editor was applied.*
Authentication	*Describe any authentication procedures for each seed stock used or novel genotype generated. Describe any experiments used to assess the effect of a mutation and, where applicable, how potential secondary effects (e.g. second site T-DNA insertions, mosiacism, off-target gene editing) were examined.*

Magnetic resonance imaging

Experimental design

Design type	*Indicate task or resting state; event-related or block design.*
Design specifications	*Specify the number of blocks, trials or experimental units per session and/or subject, and specify the length of each trial or block (if trials are blocked) and interval between trials.*

Behavioral performance measures

State number and/or type of variables recorded (e.g. correct button press, response time) and what statistics were used to establish that the subjects were performing the task as expected (e.g. mean, range, and/or standard deviation across subjects).

Acquisition

Imaging type(s)

Specify: functional, structural, diffusion, perfusion.

Field strength

Specify in Tesla

Sequence & imaging parameters

Specify the pulse sequence type (gradient echo, spin echo, etc.), imaging type (EPI, spiral, etc.), field of view, matrix size, slice thickness, orientation and TE/TR/flip angle.

Area of acquisition

State whether a whole brain scan was used OR define the area of acquisition, describing how the region was determined.

Diffusion MRI ☐ Used ☐ Not used

Preprocessing

Preprocessing software

Provide detail on software version and revision number and on specific parameters (model/functions, brain extraction, segmentation, smoothing kernel size, etc.).

Normalization

If data were normalized/standardized, describe the approach(es): specify linear or non-linear and define image types used for transformation OR indicate that data were not normalized and explain rationale for lack of normalization.

Normalization template

Describe the template used for normalization/transformation, specifying subject space or group standardized space (e.g. original Talairach, MNI305, ICBM152) OR indicate that the data were not normalized.

Noise and artifact removal

Describe your procedure(s) for artifact and structured noise removal, specifying motion parameters, tissue signals and physiological signals (heart rate, respiration).

Volume censoring

Define your software and/or method and criteria for volume censoring, and state the extent of such censoring.

Statistical modeling & inference

Model type and settings

Specify type (mass univariate, multivariate, RSA, predictive, etc.) and describe essential details of the model at the first and second levels (e.g. fixed, random or mixed effects; drift or auto-correlation).

Effect(s) tested

Define precise effect in terms of the task or stimulus conditions instead of psychological concepts and indicate whether ANOVA or factorial designs were used.

Specify type of analysis: ☐ Whole brain ☐ ROI-based ☐ Both

Statistic type for inference

(See Eklund et al. 2016)

Specify voxel-wise or cluster-wise and report all relevant parameters for cluster-wise methods.

Correction

Describe the type of correction and how it is obtained for multiple comparisons (e.g. FWE, FDR, permutation or Monte Carlo).

Models & analysis

n/a	Involved in the study
☐	☐ Functional and/or effective connectivity
☐	☐ Graph analysis
☐	☐ Multivariate modeling or predictive analysis

Functional and/or effective connectivity

Report the measures of dependence used and the model details (e.g. Pearson correlation, partial correlation, mutual information).

Graph analysis

Report the dependent variable and connectivity measure, specifying weighted graph or binarized graph, subject- or group-level, and the global and/or node summaries used (e.g. clustering coefficient, efficiency, etc.).

Multivariate modeling and predictive analysis

Specify independent variables, features extraction and dimension reduction, model, training and evaluation metrics.
